# Functional Cortical Network in Alpha Band Correlates with Social Bargaining

**DOI:** 10.1371/journal.pone.0109829

**Published:** 2014-10-06

**Authors:** Pablo Billeke, Francisco Zamorano, Mario Chavez, Diego Cosmelli, Francisco Aboitiz

**Affiliations:** 1 División Neurociencia de la Conducta, Centro de Investigación en Complejidad Social (CICS), Facultad de Gobierno, Universidad del Desarrollo, Santiago, Chile; 2 Centro Interdisciplinario de Neurociencias, Pontificia Universidad Católica de Chile, Santiago, Chile; 3 Departamento de Psiquiatría, Escuela de Medicina, Pontificia Universidad Católica de Chile, Santiago, Chile; 4 Escuela de Psicología, Pontificia Universidad Católica de Chile, Santiago, Chile; 5 CNRS UMR-7225, Hôpital de la Salpêtrière, Paris, France; Hospital for Sick Children, Canada

## Abstract

Solving demanding tasks requires fast and flexible coordination among different brain areas. Everyday examples of this are the social dilemmas in which goals tend to clash, requiring one to weigh alternative courses of action in limited time. In spite of this fact, there are few studies that directly address the dynamics of flexible brain network integration during social interaction. To study the preceding, we carried out EEG recordings while subjects played a repeated version of the Ultimatum Game in both human (social) and computer (non-social) conditions. We found phase synchrony (inter-site-phase-clustering) modulation in alpha band that was specific to the human condition and independent of power modulation. The strength and patterns of the inter-site-phase-clustering of the cortical networks were also modulated, and these modulations were mainly in frontal and parietal regions. Moreover, changes in the individuals’ alpha network structure correlated with the risk of the offers made only in social conditions. This correlation was independent of changes in power and inter-site-phase-clustering strength. Our results indicate that, when subjects believe they are participating in a social interaction, a specific modulation of functional cortical networks in alpha band takes place, suggesting that phase synchrony of alpha oscillations could serve as a mechanism by which different brain areas flexibly interact in order to adapt ongoing behavior in socially demanding contexts.

## Introduction

In daily life, we spend an great deal of time dealing with social dilemmas [1]. A crucial characteristic of such situations is that goals that tend to clash can co-exist with the consequence of making an analytical approach non-trivial [2]. Naturally, then, when people face social dilemmas, several cognitive processes must be recruited. Neurobiological studies have identified several brain areas which underlie different functions supporting our capacity to maintain a social interaction and solve social dilemmas [3], [4]. Rather than having specific and isolated functions, it has been proposed that these areas work as a network which requires a rapid, efficient, adaptive interaction among them and with other domain-general networks [5], [6]. Thus social processing, like empathy [7] and imitation [8], has been shown to generate an increase in the connectivity among different brain areas. In spite of this evidence, it remains unknown whether specific changes in functional connectivity of the brain networks are related to social behavior. Indeed, a recent study shows that flexible organization in connectivity patterns of fronto-parietal network is related to our capacity to adapt our cognitive resources according to the task demands [9]. Thus, we hypothesize that during social interactions, a particular functional reorganization of brain functional connectivity takes place, and that this reorganization is reflected in the dynamics of the cortical networks as estimated by inter-site-phase-clustering between brain sources.

**Figure 1:**
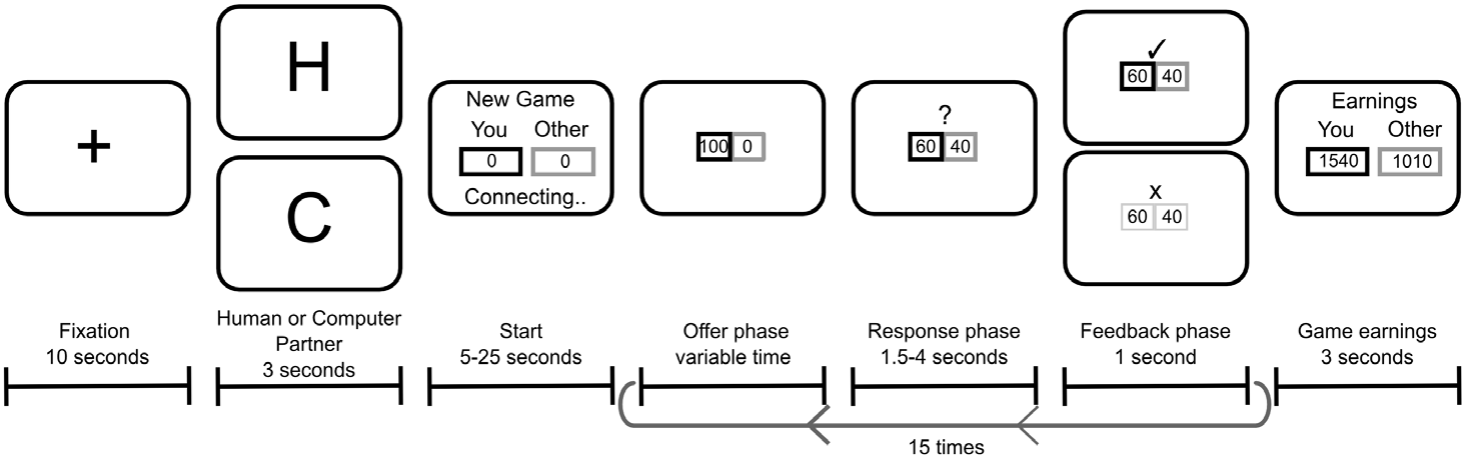
Timeline of a game. Proposers (black box) and responders (gray box, computational simulations, see Methods) played a repeated Ultimatum Game. The proposer makes an offer on how to split 100 Chilean pesos between the responder and himself/herself (offer phase). The responder decides to either accept or reject it (response phase). If the responder accepts the offer, the money is split as proposed, and if he/she rejects it, the money is lost. The response is shown on the screen during 1 s (feedback phase). Each game consists of 15 offers. At the beginning of each game, the proposer sees a cue that indicates if his/her partner is a human (“H”) or computer (“PC”).

Empirical studies have led to the hypothesis that functional neural assemblies are largely distributed and linked to form a web-like structure in the brain [10]. Within this framework, brain regions are conceived as partitioned into a collection of modules, representing functional units that are separable from -but related to- the functions of other modules. Detecting the modular brain structure may be crucial to understanding the structural and functional properties of neural systems during social interactions. To evaluate this possibility, we used Graph Theory analysis of the electroencephalographic (EEG) activity of human subjects while they played a standard behavioral economics game that recreates a social dilemma of bargaining, namely the repeated version of the Ultimatum Game (Figure 1) [11], [12]. The game involves two players, namely the proposer and the responder. The proposer makes an offer as to how to split a certain amount of money between the two players. The responder can either accept or reject the offer. If the offer is accepted, the money is split as proposed, but if it is rejected, neither player receives any money. Crucially, during this repeated interaction, proposers have to predict the most probable behavior of the responders to estimate the risk of their actions and adapt their own behavior accordingly [13]. To make this behavioral prediction, it is necessary to recruit brain networks that participate in social processing to figure out the other players’ intentions (e.i., Mentalizing) [14].

## Methods

Twenty-five individuals (11 women) participated for monetary compensation after online recruitment. Seventeen subject data were originally recollected for the control task of a previous work [15]. All the analyses presented here are new. All participants were right-handed Spanish speakers, aged from 20 to 37 years (M = 25.31, SEM = 0.71). All participants had normal or corrected-to-normal vision, no color-vision deficiency, no history of neurological diseases, and no current psychiatric diagnosis or psychotropic prescriptions. All participants provided their written informed consent to participate in this study and the Ethics Committee of the Pontificia Universidad Católica de Chile approved the experimental protocol. All experiments were performed at the Cognitive Neuroscience Laboratory of the Department of Psychiatry of the University.

### Task

Participants played as proposers in a repeated version of the Ultimatum Game (see Figure 1). Subjects believed they were playing either with a human partner or a computer partner, but they were actually always playing with a computational simulation (see below). Participants began their participation by reading on-screen instructions describing the game. At the beginning of each game, participants watched the fixation cross (10 seconds, fixation phase). Then, a signal on the screen indicated whether the game was against a computer (“PC”) or a human (“H”) partner. Each game consisted of 15 rounds and each participant played as a proposer 32 times with different simulated responders (16 human games and 16 computer games, randomly distributed). For computer games, the experimenter explained to the participant that the computer simulation assigned a probability to accept the offer given the amount of money offered (a direct relation), and that this probability could change between different games but not during a game with the same computer partner. Importantly, the simulation used in human and computer games was the same. Each trial had three phases as follows: In the first (offer phase, variable duration), the proposer had to make the offer. In the second (anticipation phase, 1.5–4 seconds), the proposer waited for the response of the partner. In the last phase (feedback phase, 1 second), the response was revealed to the proposer. At the end of each game, the earnings each player had made in the game were revealed. After the set of games concluded, the experimenter interviewed each participant individually in order to check whether they had understood the game correctly. The amount of money each participant received depended on his/her performance in one of the 32 games chosen randomly, and ranged from 6,000 to 12,000 Chilean pesos, 12 to 24 USD approximately.

### Simulation

Simulations used in the tasks were based on a mixed logistic modeling of 33 people playing as receptors with other people (for more details see [13]). Using this model, we were able to create different virtual players. All participants played with the same simulated partners. Specifically, the simulation algorithm assigns a probability to reject or accept the offer given the following two equations:

for round (x) = 1,
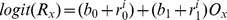



and for round (x) >1,
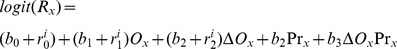
where 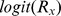 is the logit transform of the probability of rejection for the round x, O_x_ the offer, ΔO_x_ the change of offer in relation to the preceding offer, and PR_x_ the preceding response. The coefficients for each regressor were composed by a population parameter (b_y_) and a random effect for each simulated responder (r_y_^i^, y = regressor and i = simulated partner). The simulation and experimental setting generated a credible human interaction for the following reasons: (1) The distributions of acceptances and rejections, and the offering behaviors related to a rejection in the simulation game were similar to those obtained in a real human game [13], suggesting that simulated responders elicited comparable behaviors in proposers. (2) During post hoc interviews, experimenters asked participants whether they believed that they had played against a human counterpart. All participants indicated that they actually believed that they had played against another human and that they felt the human games different from the computer games. We used the 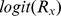 to the quantification of the risk per each offer made.

### Electrophysiological Recordings

Continuous EEG recordings were obtained with a 40-electrode NuAmps EEG System (Compumedics Neuroscan). All impedances were kept below 5 kΩ. Electrode impedance was retested during pauses to ensure stable values throughout the experiment. All electrodes were referenced to averaged mastoids during acquisition and the signal was digitized at 1 kHz. Electro-oculogram was obtained with four electrodes with both vertical and horizontal bipolar derivation. All recordings were acquired using Scan 4.3 (Compumedics Neuroscan) and stored for off-line treatment. At the end of each session, electrode position and head points were digitalized using a 3D tracking system (Polhemus Isotrak).

### EEG Data Analysis

EEG signals were preprocessed using a 0.1–100 Hz band-pass Butterworth filter (third-order, forward and reverse filtering). Eye blinks were identified by a threshold criterion of ±100 µV, and their contribution was removed from each dataset using principal component analysis by singular value decomposition and spatial filter transform using Scan 4.3 (Compumedics Neuroscan). Other remaining artifacts (e.g., muscular artifacts) were detected by visual inspection of the signal and the trials that contained them were removed. After this procedure, we obtained 424±33 artifact-free trials across the subjects. Time frequency (TF) distributions were obtained by means of the wavelet transform, between –1.5 and 1.5s. We displayed the result only for –1 to 1 s over the segmented signals to avoid edged artifact. A signal x(t) was convolved with a complex Morlet’s wavelet function defined as 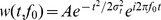. Wavelets were normalized and thus 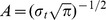 the width of each wavelet function 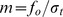 was chosen to be 7; where 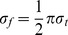. TF contents was represented as the energy of the convolved signal: 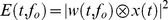. Thus, we obtained the phase and amplitude per each temporal bin (in steps of 10 ms) and frequency (from 4 to 30 Hz in step of 1 Hz). We used for analysis only the 90 riskiest and the 90 safest offers per subject and condition, in order to ensure equal number of trials for statistical comparison. For all power spectrum analysis, we used the dB of power related to a baseline during the fixation phase (ten seconds at the beginning of each game, Figure 1).

### Source Estimations

The neural current density time series at each elementary brain location was estimated by applying a weighted minimum norm estimate inverse solution [16] with unconstrained dipole orientations in single-trials. A tessellated cortical mesh template surface derived from the default anatomy of the Montreal Neurological Institute (MNI/Colin27) wrapped to the individual head shape (using ∼300 headpoints per subject) was used as a brain model to estimate the current source distribution. We defined 3×390 sources constrained to the segmented cortical surface (3 orthogonal sources at each spatial location, avoiding deep and basal structures since the sensitivity of the EEG signal to the activity of those structures is poor), and computed a three-layer (scalp, inner skull, outer skull) boundary element conductivity model and the physical forward model [17]. The measured electrode level data 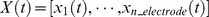 is assumed to be linearly related to a set of cortical sources 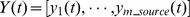 and additive noise 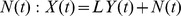, where L is the physical forward model. The inverse solution was then derived as 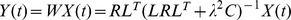 where W is the inverse operator, R and C are the source and noise covariances respectively, the superscript T indicates the matrix transpose, and λ^2^ is the regularization parameter. R was the identity matrix that was modified to implement depth-weighing (weighing exponent: 0.8 [18]), The regularization parameter λ was set to 1/3. To estimate cortical activity at the cortical sources, the recorded raw EEG time series at the sensors x(t) were multiplied by the inverse operator W to yield the estimated source current, as a function of time, at the cortical surface: 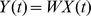. Since this is a linear transformation, it does not modify the spectral content of the underlying sources. It is therefore possible to undertake time–frequency analysis on the source space directly. Finally, we reduced the number of sources by keeping a single source at each spatial location that pointed into the direction of maximal variance. To this end, we applied a principal component analysis to covariance matrix obtained from the 3 orthogonal time series estimated at each source location. This resulted in a single filter for each spatial location that was then applied to the complex valued data to derive frequency specific single trial source estimates. Since we used a small number of electrodes (40) and no individual anatomy for head model calculation, the spatial precision of the source estimations are limited. In order to minimize the possibility of erroneous results we only present source estimations if there are both statistically significant differences at the electrode level and the differences at the source levels survive a multiple comparison correction.

### Functional Network

We consider the functional links in brain signals defined via the phase-locking value (PLV) computed between all pairs of electrodes or brain sources [19]. The PLV measures the inter-site-phase-clustering. To compute the PLVs, we used a complex Morlet’s wavelet function of 7 cycles. By means of this complex wavelet transform, an instantaneous phase 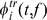 is obtained for each frequency component of signals i (electrodes or sources) at each trial (tr). The PLV between any pair of signals (i,k) is inversely related to the variability of phase differences across trials:
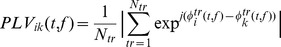
where N_tr_ is the total number of trials. If the phase difference varies little across trials, its distribution is concentrated around a preferred value and PLV<1. In contrast, under the null hypothesis of a uniformity of phase distribution, PLV values are close to zero. Finally, to assess whether two different nodes are functionally connected, we calculated the significance probability of the PLVs by a Rayleigh test of uniformity of phase. According to this test, the significance of a PLV determined from N_tr_ can be calculated as 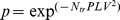 [20]. To correct for multiple testing, the False Discovery Rate (FDR, q<0.05) method was applied to each matrix of PLVs. In the construction of the networks, a functional connection between two brain sites was assumed as an undirected and weighted edge (functional connectivity strength between node w_ij_ = PLV_ij_, for significant links and w_ij_ = 0 otherwise). We calculated the strength of inter-site-phase-clustering for each node (electrode or source) as the sum of all significant PLVs of that node.

### Network partitions

To partition the functional networks in modules, we used a random walk-based algorithm [21]. This data-driven approach is based on the intuition that a random walker on a graph tends to remain into densely connected subsets corresponding to modules. To find the modular structure, the algorithm starts with a partition in which each node in the network is the sole member of a module. Modules are then merged by an agglomerative approach based on a hierarchical clustering method. At each step the algorithm evaluates the quality of partition 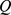, which compares the abundance of edges lying inside each community with respect to a null model. The modularity of a given partition is defined as, 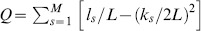 where M is the number of modules, L is the total number of connections in the network, 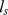 is the number of connections between vertices in module s, and 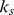 is the sum of the degrees of the vertices in modules. The partition that maximizes 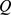 is considered as the partition that better captures the modular structure of the network. Further details can be found in [22], [23].

To evaluate the agreement between community structures we use the Rand index [24], which is a traditional criterion for comparison of different results provided by classifiers and clustering algorithms, including partitions with different numbers of classes or clusters. For two partitions P and P’, the Rand index is defined as 
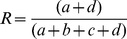
; where 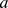 is the number of pairs of data objects belonging to the same class in P and to the same class in P’, 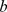 is the number of pairs of data objects belonging to the same class in P and to different classes in P’, 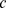 is the number of pairs of data objects belonging to different classes in P and to the same class in P’, and 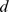 is number of pairs of data objects belonging to different classes in P and to different classes in P’. The Rand index has a straightforward interpretation as a percentage of agreement between the two partitions and it yields values between 0 (if the two partitions are randomly drawn) and 1 (for identical partition structures).

### Statistical analysis

For pair comparison and correlation, we used non-parametric tests (Wilcoxon and Spearman correlation). For multiple regressions, we used robust linear regression. To correct for multiple comparisons in time-frequency chart and sources, we used the Cluster-based permutation test for the EEG data [25]. In the latter method, clusters of significant areas were defined by pooling neighboring sites (in the time-frequency chart) that showed the same effect (p<0.05). The cluster-level statistics was computed as the sum of the statistics of all sites within the corresponding cluster. We evaluated the cluster-level significance under the permutation distribution of the cluster that had the largest cluster-level statistics. The permutation distribution was obtained by randomly permuting the original data. After each permutation, the original statistical test was computed (e.g., Wilcoxon), and the cluster-level statistics of the largest cluster resulting was used for the permutation distribution. After 1,000 permutations, the cluster-level significance for each observed cluster was estimated as the proportion of elements of the permutation distribution greater than the cluster-level statistics of the corresponding cluster.

### Software

All behavioral statistical analyses were performed in R. The EEG signal processing was implemented in MATLAB using in-house scripts (LAN toolbox, available online at http://lantoolbox.wikispaces.com/, e.g. [26]). For the source estimation and head model, we used the BrainStorm [27] and openMEEG toolboxes [28].

## Results

### Behavior

Subjects in both human (HGs) and computer games (CGs) made comparable offers in the amount of money (HG = $42.5; CG = $42.3, Chilean pesos; Wilcoxon signed rank test; p = 0.78) and risk (measured as the logit of the probability to acceptance; HG = 0.89; CG = 0.86; p = 0.9). Like in our previous work, we found a strategic difference between HGs and CGs given by the evolution of the offer risk during a game. In CGs there was a stronger correlation between the offer risk and the round number (Spaerman’s rho = 0.88, p<2e−16) than that of HG (rho = 0.61, p = 0.01), giving a difference in the interaction between conditions (HG and CG) and round number in the robust linear regression (Table 1). These results suggest that subjects use a learning strategy in CGs but a bargaining strategy in HGs [15].

**Table 1:** Model of the risk of offers.

	Scope	Std. Error	T-value	p-value
(Intercept)	0.3747	0.1136	3.2976	0.00002
**Round**	0.1021	0.0125	8.1701	0.00000001
**Human Games**	0.3101	0.1607	1.9296	0.06
**Round×Human Games**	−0.0686	0.0177	−3.8813	0.0006
degree of freedom = 26				

### EEG

**Figure 2:**
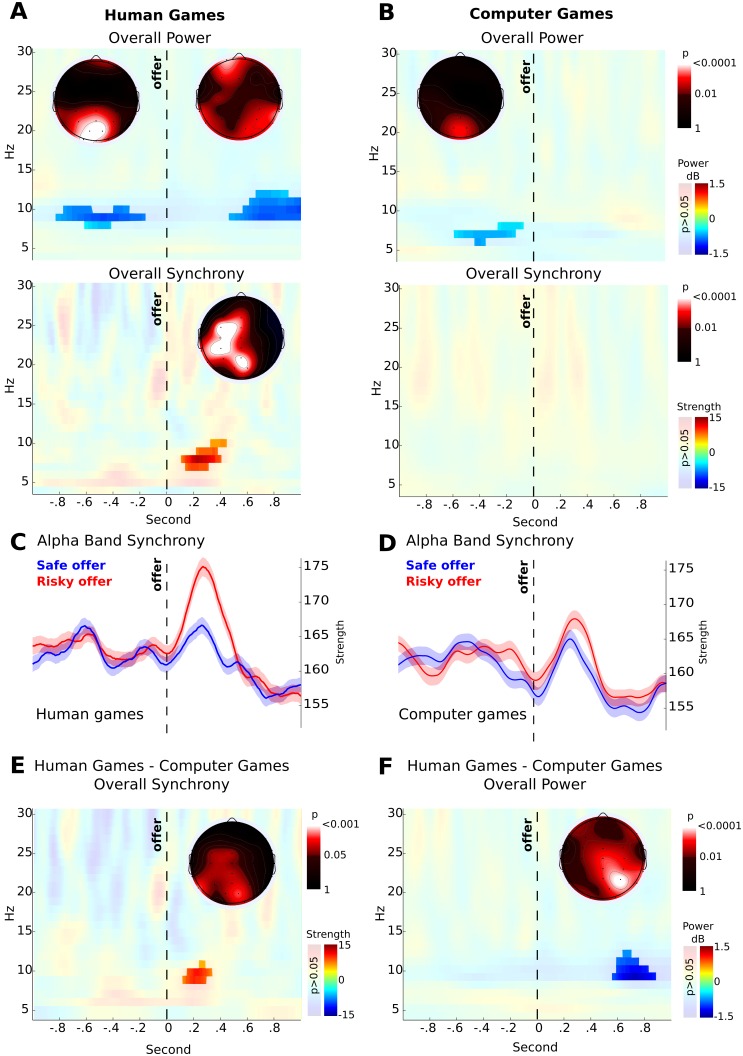
Scalp levels. **A, B,** Time-frequency charts of the difference between risky and save offers in human (**A**) and computer games (**B**). The upper panel shows differences in the overall power (dB) and the lower panel in strength of synchrony (inter-site-phase-clustering). **C, D** Inter-site-phase-clustering strength in alpha band (7–10 Hz) in safe (blue) and risky (red) offer in human (**C**) and computer (**D**) games. Areas represent the standard error of means. **E, F,** Difference between human and computer games in overall power (**E**) and synchrony (**F**). **A–F,** The non-significant areas are overshadowed and for each significant area, the scalp distribution of p-values is shown. Time-frequency charts and time line plots show the mean of the power or the sum of inter-site-phase-clustering strengths across all electrodes, and the topographic plots show the distribution of the significant time-frequency windows highlighted.

We explored the oscillatory brain activity related to the anticipation of the other’s response. We calculated the risk for each offer and compared the 90 riskiest with the 90 safest offers per subject. To explore changes in the global dynamics we first compared the overall power and inter-site-phase-clustering strength (by means of PLV) between risky and safe offers per condition, at the electrode level. For this, we explored for changes in the sum of the inter-site-phase-clustering strength or power across all electrodes. First we defined a time-window of interest (0 to 1 second after the subject made the offer) and calculated a repeated measure ANOVA. In this analysis we found that the interaction between conditions (Human and Computer games), offer risk (risky vs. safe offer) and frequency band (theta, 4–7 Hz, alpha, 8–12 Hz, beta 13–25 Hz) was significant (F_2_ = 3.25, p = 0.0406, Table S1 in File S1). Then, we explored the entire time-frequency chart and, in those regions where we found significant modulations, we explored their topographies and the electrodes that showed significant effect (Figure 2).

Over occipital electrodes, we found a significant drop in alpha power before the subject made the offer in both HGs and CGs (main effect: 8–10 Hz; –0.8 to –0.2 s; O1 and O2 electrodes; Figure 2A–B). After the subjects made the offer and before they received the response (anticipatory phase), we found a drop in alpha band power over left posterior temporo-parietal electrodes only in HGs (main effect: 9–12 Hz; 0.5 to 1 s; TP8 electrode; Figure 2A, upper panel, Wilcoxon rank sum test and cluster based permutation test, p<0.01). During this anticipatory phase, we also found an increase of the alpha inter-site-phase-clustering strength prior to the difference in power (main effect: 7–10 Hz; 0.2 to 0.4 s; FC3, C3, CP3 and Pz electrode; Figure 2A, lower panel, note that the inter-site-phase-clustering strength was calculated based on all possible electrode pairs). This synchrony increase was mainly in risky offers made during HG (7–10 Hz, Figure 2C). Notably, we did not find any difference in inter-site-phase-clustering between risky and safe offers made during CGs (Figure 2B–D).

**Figure 3:**
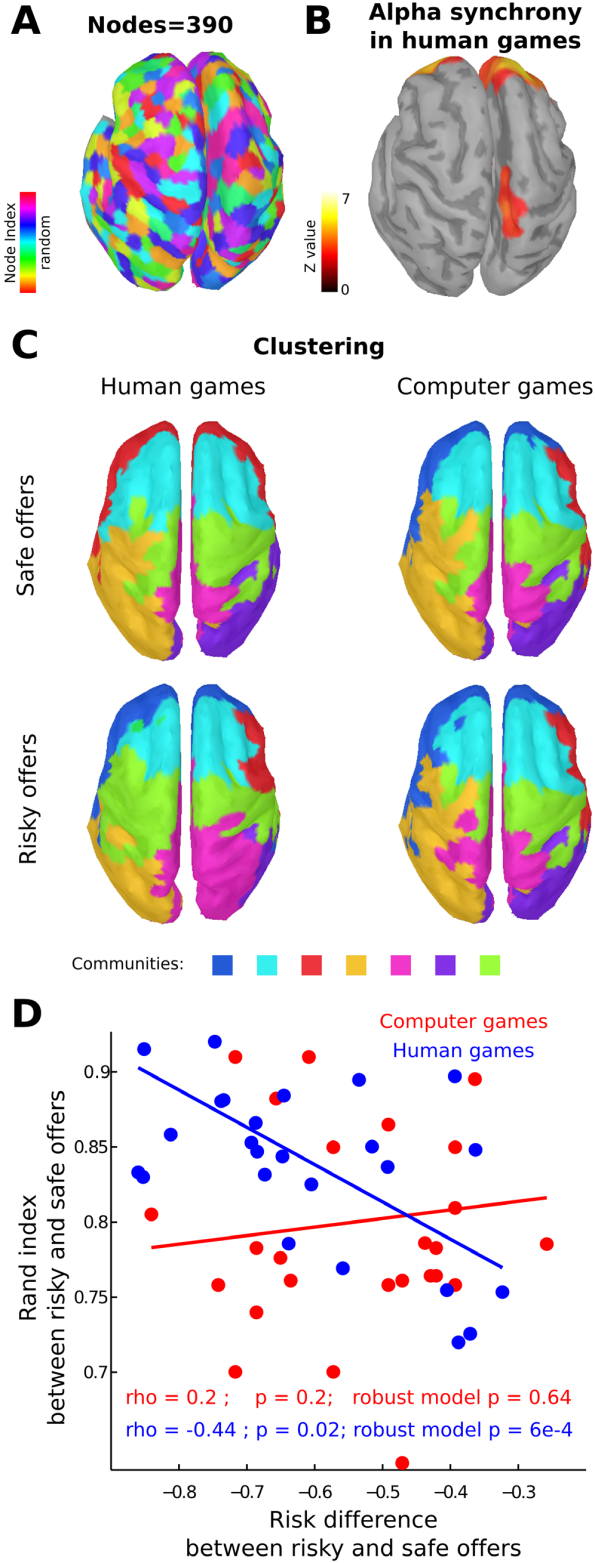
Source level. **A,** Cortical areas that represent each node in the global network (colors represent node indexes in random order). **B,** Areas where the synchrony (inter-site-phase-clustering) differences between risky and safe offers in human game were significant (FDR, q<0.05). **C,** Community structure of the population networks by conditions (colors represent de community index). Note that there are no main variations between risky and safe offers in the computer game, while there is a notorious variation in human games. **D,** Correlation between the differences in the risk of the offers made (logit of the probability to acceptance, risky offers – safe offers) and the change of the community structure per each subject. Note that only in human games there is a significant correlation. Blue depicts human games and red computer games.

Since the volumetric conduction of distal sources can spuriously generate synchrony at the electrode level, we carried out the same analysis using both the current source density (CSD) at the electrode level and source reconstruction at cortical level. CSD analysis replicates the difference between human and computer games shown in Figure 2 (See Figure S1 in File S1). For source reconstruction, we calculated the electrical activity in 390 source nodes over the cortex (Figure 3A), avoiding subcortical structures where the sensitivity of EEG is poor. Then, we calculated the strength of inter-site-phase-clustering for each node in alpha band (7–10 Hz, where we found the main modulation at the electrode level), and found differences in the medial parietal and frontal nodes between safe and risky offers for HGs but not for CGs (Figure 3B). In order to evaluate if the strength change reflects a change in functional network, we calculated the community structure of the networks at group and individual levels for both conditions at cortical source level (see Methods).

In agreement with the above result, we found that the community structure changed between risky and safe offers in HGs but not in CGs, at group levels, in frontal and parietal regions (Figure 3C). We obtained seven communities per condition except for safe offers in HGs in which we obtained six. We found a bilateral module in dorsolateral prefrontal cortex, which was conserved across conditions (light-blue in Figure 3C). We observed two modules in inferior frontal gyrus and fronto-polar regions, which were joined only in safe offers in HGs (blue and red in Figure 3C). We also found a central module in sensory-motor cortex, which was greater especially in the left hemisphere in risky offers in HGs (Green). Finally, we detected one medial and two lateral modules in parietal and occipital regions, which changed in risky offers in HGs in comparison with the other conditions.

In order to evaluate whether this differential functional organization is specifically related to social interaction, we computed the individual differences in community structure between risky and safe offer networks using the rand index (at the cortical source level). Interestingly, the difference in community structure was significantly correlated with the differences in the risk of the offer only in HGs (rho = –0.44, p = 0.02), but not in CGs (rho = 0.2, p = 0.2). Indeed, using robust linear regression, the interaction between conditions (HGs and CGs) and risk differences was significant (t_46_ = –2.05, p = 0.045) even after correcting by power and inter-site-phase-clustering strength differences between risky and safe offers (Table 2). This indicates that functional network activation was specifically related to the behavior during HGs (t_44_ = –2.42, p = 0.02) independently of change of strength of inter-site-phase-clustering (p = 0.99) and possible influences of changes in power (p = 0.04, Table 2).

**Table 2:** Model of the difference in modularity between risky and safe offer (Rand Index).

	Scope	Std. Error.	T-value	p-value
(Intercept)	0.8329	0.0539	15.4663	0
**Risk diff.**	0.0639	0.0939	0.6801	0.5008
**Strength diff.**	0	0.0003	–0.0093	0.9926
**Power diff.**	0.5665	0,268	2.1138	0.0415
**Human Games**	–0.128	0.0711	–1.7992	0.0804
**Risk diff.×Human Games**	–0.2923	0.1203	–2.4295	0.0202
degree of freedom = 44				

## Discussion

It has been proposed that the complex and flexible behaviors that sustain human social interactions rely on the dynamic modulation of patterns of interaction among specialized large-scale brain systems [29]. Here, we explored such specific modulations of patterns in brain connectivity during two types of interaction. Crucially, we used two tasks that were exactly the same except by the context instructed to the subject (human *vs* computer partners), and we found a significant modulation in alpha power and inter-site-phase-clustering, depending on the social context. A recent work has shown that inter-site-phase-clustering in alpha band at the electrode level correlates with the activity of fronto-parietal networks [30]. In that work, using concomitant EEG and fMRI recordings during rest, the authors found that the inter-site-phase-clustering of alpha band was specifically correlated with the BOLD signal of frontal pole, inferior parietal lobe and medial parietal lobe [30]. Interestingly, the activity of fronto-parietal networks is specifically correlated to alpha band, and does not show correlation with other frequency bands [30]. This specific correlation includes the anterior prefrontal cortex and the medial parietal cortex where we found inter-site-phase-clustering strength modulation at source level. Although other studies have found a correlation between alpha inter-site-phase-clustering with default mode network (see [31]), our results are in accordance with the correlation with fronto-parietal regions (compared Figure 2B with Figure 1 in [30]), and match the hypothesis that the fronto-parietal network participates in cognitive control, especially in a trial-by-trial high adaptive control situation [32]. Moreover, evidence from intracortial recording in monkeys, shows the existence of a prefronto-parietal network that shows phase synchrony at in 5–10 Hz [33]. This network increases its inter-site-phase-clustering strength during the anticipation of top-down controlled processes [33]. Indeed, cognitive control is highly required to solve difficult social situations like the dilemmas recreated by game theory tasks such as the one used here [34]. In the same line, it has been proposed that cognitive control is necessary for humans to develop pro-social behavior like mutual cooperation [35]. Thus, the increase in phase synchrony that we found probably reflects the higher cognitive demands required by the expectation of the partner’s behavior in a repeated interaction with humans (e.g., integrating the other’s intention, the previous interactions and the future consequences) than that required in a computer interaction.

An important limitation of our work concerns the interpretation of functional connectivity using EEG. Volume conduction may cause spurious connectivity by the fact that activity in one source can be represented in multiple measurement points [36]. In order to lessen erroneous results, we explored the synchrony in both electrode and source reconstruction levels, and studied global dynamics rather that local modulations. Additionally, our results indicated that inter-site-phase-clustering changes were dissociated from power changes, which argues against possible volume conduction effects. Finally, behavior was significantly related to network dynamics independently of its overall inter-site-phase-clustering strength and power.

It has been proposed that alpha power shows a negative correlation with cortical activity [37]–[40]. Alpha power has shown a negative correlation with the dorsal attention network and a positive correlation with the cingulo-opercular network, with no relation to the fronto-pariental network [41]. Works in focused attention suggest that phase locking during the processing of a stimulus can occur with concomitant amplitude reduction [42]. Additionally, it seems that oscillatory alpha activity operates in a phasic manner [43], [44]. Thus, the phase of pre-stimulus alpha oscillations modulates visual detection [45]. Following this evidence, it has been proposed that alpha oscillation works as a pulsed inhibition and that its synchronized activity could have a important role in the change of network activity in the brain [46]–[48]. In our experiment, alpha inter-site-phase-clustering modulation was temporally dissociated from power modulation (Figure 2). Using the same task but only considering games among actual human partners, we have previously shown that the power modulation in alpha is associated with the risk of the offer in a temporo-parietal region compatible with social processing areas (such as the temporo-parietal junction and superior temporal sulcus), and the entropy of the offer in regions syndicated as part of the cingulo-opercular network [13]. Altogether, this evidence is compatible with a functional dissociation between phase and power modulation in the alpha band measure over the scalp [49].

Since cognitive control processes necessary to solve social dilemmas require flexible long-range communication and integration among brain areas [5], we finally explored changes in the cortical network structure using graph theory analysis. At the population level, the community structure between risky and safe offers did not differ in games with computer partners. Interestingly, in human games the community structure was different between conditions mainly in prefrontal and parietal cortex. Although a one-to-one assignment of anatomical brain regions to the retrieved modules is difficult to define, our results reveal an overlap between the modules and some well-known functional areas of the brain. The modules in inferior frontal gyrus are compatible with the frontal parts of the fronto-parietal control network [50]. As we mentioned above, cognitive control is an essential process that enables us to carry out social interaction [34]. In childhood, prefrontal cortex maturation correlates with both impulse control and cooperative behavior in social contexts [51]. In addition, alterations in cognitive control are an important factor underlying social impairments of several psychiatric diseases such as schizophrenia [52], [53] and depression [54], [55]. The notorious differences in frontal and parietal modules in HGs may therefore reflect the behavioral control required to carry out a social bargaining. Across the HGs, people tended to change less their offers, as if expecting that the others would also change their behaviors (Table 1, and [15]). Thus on the first rounds, people can tolerate rejection and do not change their behavior in order to obtain more acceptances on the later rounds [15], [56]. However, to maintain this strategy, a greater cognitive control is required. In accordance with this interpretation, we found a specific correlation in HGs, where people who presented less difference between high-risk and low-risk offers showed a greater functional network differentiation (Figure 3D).

The functional networks that we found also include a central module in sensory-motor cortex. This module is compatible with the source of mu rhythms [57] whose power decreases (also called suppression or local desynchronization) has been related to mirror system activity during social games [58], motor coordination between humans [59] and the imagination of social interactions [60]. Moreover, recent work has shown that power in alpha range over sensory motor cortex is related to facial emotion recognition and it is negatively correlated with connectivity of the motor sensory cortex with other cortical areas [61]. In our experiment we found that the main modulation of global alpha synchrony was temporally dissociated from power modulation (Figure 2). However, the change in power was related to the community structure and not to the overall synchrony as the robust linear model shows (Table 2). This most likely reflects that the relations between phase synchrony and power is specific for each cortical area. Due to the low spatial resolution of our source reconstructions, we cannot accurately test these local differences. Finally, the spatial variations of the modules in lateral parietal region could be related to attentional and control networks that present nodes in this region [62], [63]. Thus, the variation of community structure over these networks could reflect flexible changes in attentional and control processing during social interactions.

In conclusion, our results complement evidence that shows that during social interaction, humans establish flexible global interactions among different brain regions [6]. More specifically, we show that changes in the functional network in alpha band range takes place during social interaction, which could reflect cognitive control requirements to maintain an ongoing bargaining. Inter-site-phase-clustering (phase synchrony) of alpha oscillations could therefore serve as a mechanism by which cognitive control areas exert modulation over and receive information from social specialized brain areas in order to adapt behavior to current demands and changing social contexts.

## Supporting Information

File S1 **Supporting information. Table S1 in File S1.** Analysis of variance of the modulation of the overall inter-site-phase-clustering strength by the conditions (human and computer games), offer risk (risky and safe offers) and frequency band (theta, alpha and beta). **Figure S1 in File S1.** Alpha band (7–10 Hz) inter-site-phase-clustering strength difference between safe and risky offers in human (red line) and computer (blue line) games. In the upper panel, inter-site-phase-clustering was calculated over voltage data reference to linking mastoids. In the lower panel, inter-site-phase-clustering was calculated over current source density (CSD). Grey areas indicate significant differences between human and computer games (p<0.05, cluster-corrected).
(DOCX)
