# Supplementary material for: Functional Cortical Network in Alpha Band Correlates with Social Bargaining
Source: PLoS One. 2014 Oct 6;9(10):e109829. doi: 10.1371/journal.pone.0109829 (PMC4186879; doi:10.1371/journal.pone.0109829)
Supplement: File S1 — Supporting information. Table S1 in File S1. Analysis of variance of the modulation of the overall inter-site-phase-clustering strength by the conditions (human and computer games), offer risk (risky and safe offers) and frequency band (theta, alpha and beta). Figure S1 in File S1. Alpha band (7–10 Hz) inter-site-phase-clustering strength difference between safe and risky offers in human (red line) and computer (blue line) games. In the upper panel, inter-site-phase-clustering was calculated over voltage data reference to linking mastoids. In the lower panel, inter-site-phase-clustering was calculated over current source density (CSD). Grey areas indicate significant differences between human and computer games (p<0.05, cluster-corrected). (DOCX) [file pone.0109829.s001.docx]

**Supporting Material**

**Table S1** Analysis of variance of the modulation of the overall inter-site-phase-clustering strength by the conditions (human and computer games), offer risk (risky and safe offer) and frequency band (theta, alpha and beta).

|  | d.f. | Mean Sq. | F | p -value |
| --- | --- | --- | --- | --- |
|  |  |  |  |  |
| Conditions | 1 | 3078,02 | 5,31 | 0,0222 |
| Offer Risk | 1 | 1487,56 | 2,56 | 0,1107 |
| Freq Band | 2 | 5217,86 | 9 | 0,0002 |
| Condition x Offer Risk | 1 | 434,92 | 0,75 | 0,3875 |
| Condition x Offer Risk x Freq. Band | 2 | 1885,84 | 3,25 | 0,0406 |
|  |  |  |  |  |

Conditions = Human games or Computer game, Offer Risk = High risk or Low Risk, Freq. Band = Theta (4-7 Hz), Alpha (8-12 Hz) or Beta (13-25 Hz). Time windows: 0 to 1 second after the decision.


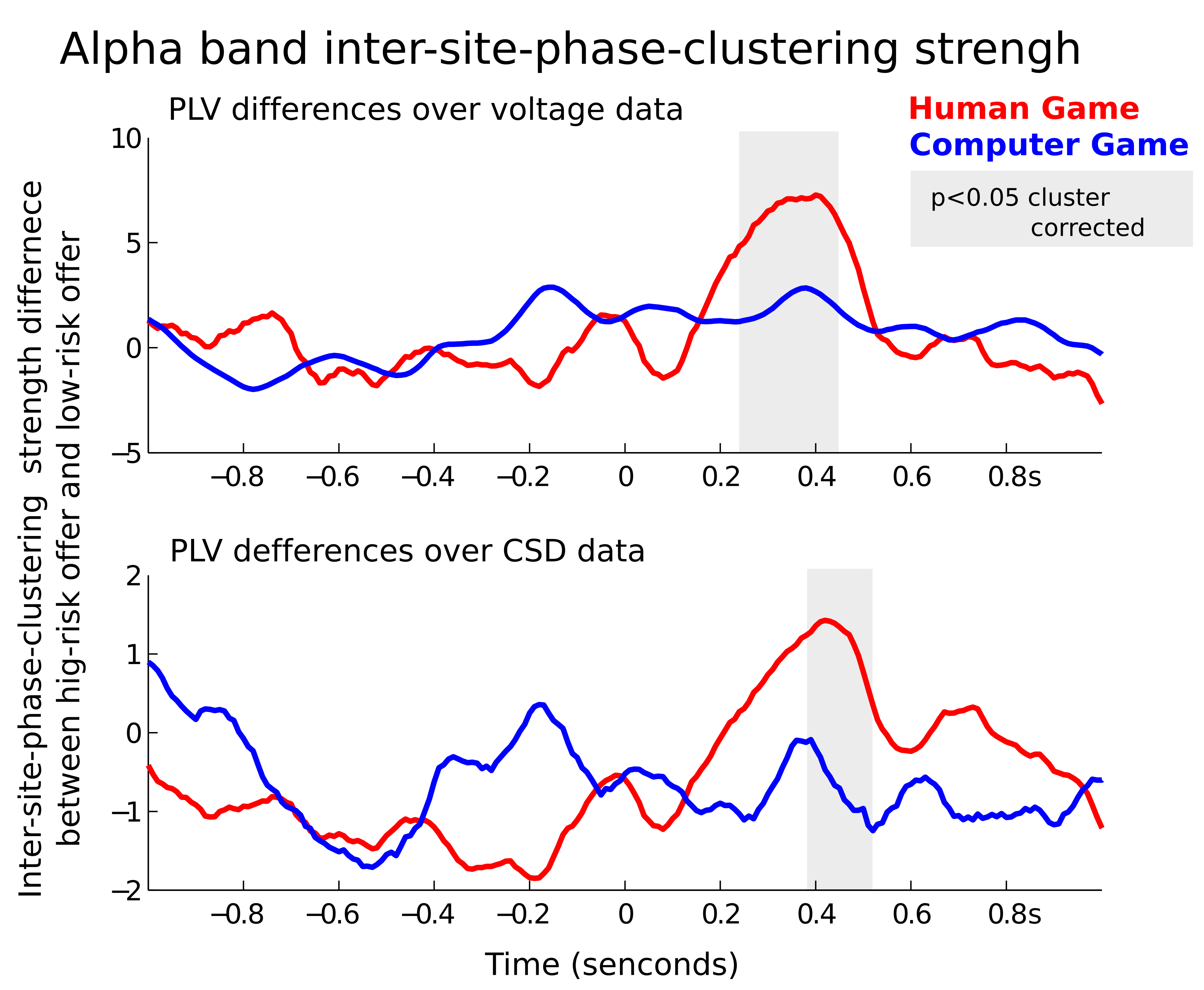


**Figure S1.** Alpha band (7 – 10 Hz) inter-site-phase-clustering strength difference between low-risk and high-risk offers in human (red line) and computer (blue line) games. In the upper panel, inter-site-phase-clustering was calculated over voltage data reference to linking mastoids. In the lower panel, inter-site-phase-clustering was calculated over current source density (CSD). Grey areas indicate significant differences between human and computer games (p<0.05m uncorrected).
